# Feasibility of assessing non-invasive intracranial compliance using FSI simulation-based and MR elastography-based brain stiffness

**DOI:** 10.1038/s41598-024-57250-4

**Published:** 2024-03-18

**Authors:** Seifollah Gholampour

**Affiliations:** Department of Neurological Surgery, The University of Chicago, 5841 S. Maryland Ave, Chicago, IL 60637 USA

**Keywords:** Intracranial compliance (ICC), Magnetic resonance elastography (MRE), Brain disorders, Brain stiffness, Viscous component, Intracranial pressure (ICP), Hydrocephalus, Computational biology and bioinformatics, Neuroscience

## Abstract

Intracranial compliance (ICC) refers to the change in intracranial volume per unit change in intracranial pressure (ICP). Magnetic resonance elastography (MRE) quantifies brain stiffness by measuring the shear modulus. Our objective is to investigate the relationship between ICC and brain stiffness through fluid–structure interaction (FSI) simulation, and to explore the feasibility of using MRE to assess ICC based on brain stiffness. This is invaluable due to the clinical importance of ICC, as well as the fast and non-invasive nature of the MRE procedure. We employed FSI simulation in hydrocephalus patients with aqueductal stenosis to non-invasively calculate ICP which is the basis of the calculation of ICC and FSI-based brain stiffness. The FSI simulated parameters used have been validated with experimental data. Our results showed that there is no relationship between FSI simulated-based brain stiffness and ICC in hydrocephalus patients. However, MRE-based brain stiffness may be sensitive to changes in intracranial fluid dynamic parameters such as cerebral perfusion pressure (CPP), cerebral blood flow (CBF), and ICP, as well as to mechano-vascular changes in the brain, which are determining parameters in ICC assessment. Although optimism has been found regarding the assessment of ICC using MRE-based brain stiffness, especially for acute-onset brain disorders, further studies are necessary to clarify their direct relationship.

## Introduction

Magnetic resonance imaging (MRI) plays a pivotal role in the visualization of the brain's structural intricacies, offering detailed insights into its anatomy. However, the traditional MRI approach has its limitations in identifying and characterizing the nuanced, diffuse abnormalities that can occur within brain tissues^1,2^. To address these limitations, magnetic resonance elastography (MRE) has been developed as an integral advancement of MRI technology. MRE is an MRI technique in which the scanner is made sensitive to motion induced by external vibrations. This refinement allows for a more comprehensive assessment of tissue characteristics, including 'softness' or 'stiffness,' providing additional, crucial insights into the physical and material properties of brain tissues that conventional MRI alone may not reveal^3,4^. Throughout the history of clinical medicine, manual examination of soft tissues in the human body using touch has been a valuable tool for detecting and characterizing pathological conditions. However, this diagnostic approach is subjective, qualitative, and limited to superficial organs and pathologies. Although there is no clinical precedent for palpating the brain directly, an analogy can be drawn between the quantitative results obtained through brain MRE and the act of palpating brain tissue. Hence, brain MRE may serve as an auxiliary tool for diagnosing certain brain disorders and providing insights into the mechanisms underlying specific conditions, such as Alzheimer's disease, Parkinson's disease, multiple sclerosis, brain tumors, normal pressure hydrocephalus (NPH), and the degenerative effects associated with healthy aging^5–10^. Evaluating brain stiffness involves applying an external force to the brain, which can be challenging due to the mechanical shielding provided by the skull, cerebrospinal fluid (CSF), and meninges. In brain MRE, mechanical vibrations are typically applied as an external force to the brain tissue, and these vibrations are encoded and detected using vibration-synchronized phase-contrast MRI^4,7,8,11,12^. This imaging technique captures the motion generated by the vibrations, allowing for the quantification of brain material properties such as stiffness, loss and storage modulus, harmonic shear strain, and dispersion using inversion algorithms^4,8^. These material properties of the brain are often crucial for investigating biological changes that deviate from the normative state^13^. Moreover, previous studies have also confirmed the significance of these material property findings obtained from brain MRE in the context of regenerative medicine^14^.

Our previous Brain MRE exhibits the potential sensitivity and capability to detect changes in interactions among brain components occurring during specific neuropathophysiological processes. Nevertheless, the potential contribution of brain MRE to ICC assessment is not fully explored. MRE is a fast and non-invasive technique, and ICC holds great clinical importance for diagnosing and evaluating treatment outcomes of brain disorders^15,16^. In addition to its clinical applications, ICC assessment can enhance our understanding of the brain's physiological and biomechanical functions, shedding light on potential implications in various neurological conditions^15^. Therefore, non-invasive ICC assessment using MRE can be highly valuable for clinical applications. The present study aims to utilize fluid–structure interaction (FSI) simulation to investigate whether brain stiffness can be used to assess ICC. Additionally, this study explores the feasibility and potential of brain MRE for ICC assessment based on brain stiffness.

### ICC and brain stiffness

ICC represents the brain's ability to buffer changes in intracranial pressure (ICP). It reflects the ability of the intracranial system to accommodate changes in intracranial volume while maintaining a stable ICP. Sustaining a stable ICP is vital to safeguard cerebral blood flow (CBF), prevent tissue harm, and maintain optimal neural activity^17^. ICC is measured as the slope of the pressure–volume curve (Eq. 1) and serves as an important indicator for predicting clinical outcomes in a wide range of brain disorders such as hydrocephalus, traumatic brain injury, intracranial hypertension, and certain types of tumors and Chiari malformations^18,19^. Previous studies have shown that the primary known methods for directly measuring ICC involve the addition or removal of fluid in the craniospinal system^19,20^; however, these experimental methods are invasive and carry inherent risks^20^. Furthermore, our recent studies have demonstrated notable variability in the methodologies of measuring ICC, highlighting concerns regarding their consistency and practical application in clinical settings^19,20^. In the present study, a non-invasive computational approach was employed to *calculate* ICC as an alternative to *measuring* it using invasive methods. The primary challenge in calculating ICC lies in determining ICP. Despite recent advances in medical imaging technology, the direct measurement of ICP through non-invasive imaging techniques remains unattainable^20,21^. Even invasive methods such as ICP monitoring do not provide comprehensive measurements of CSF pressure throughout the entire CSF circulation system. However, previous studies have employed FSI simulation method to calculate ICP and, as a result, ICC^18,21–27^. As a result, in our present study, we also employed FSI simulation as a non-invasive alternative to calculate ICP and, consequently, ICC.

1 $${\text{ICC}} = { }\frac{\Delta Volume}{{\Delta ICP}}$$

Despite being a non-invasive FSI simulation method, computer simulation methods like FSI simulations are somewhat time-consuming to calculate ICC, similar to experimental invasive ICC measurement methods^20,28^. Hence, they cannot be the first option and the most efficient method to evaluate patients with an emergency condition. Therefore, we aim to study the potential of brain stiffness and MRE-based brain stiffness as a fast and non-invasive tool to assess ICC. Brain stiffness is measured by the slope of the force–deformation curve (Eq. 2)^29^. However, MRE-based brain stiffness has a distinct unit and concept. Unlike stiffness assessments based on force and deformation using invasive experimental methods such as indentation tests or computational approaches like FSI simulation based on Eq. 2, the denominator in the equation for MRE-based stiffness incorporates the square of the wave propagation velocity. The unit of MRE-based brain stiffness, which is expressed in the shear modulus unit (Pa, denoting force per unit area, N/m^2^) also differs from brain stiffness defined as force per deformation (N/m) in Eq. 2. Therefore, we need to differentiate between brain stiffness based on force and deformation, such as FSI-based brain stiffness, and MRE-based brain stiffness.

2 $${\text{Brain stiffness}} = { }\frac{{\Delta {\text{Force exerted from CSF on the brain}}}}{\Delta Brain\, deformation} = \frac{{\Delta \left( {{\text{ICP }} \times Brain \,surface \,area} \right)}}{\Delta Brain \;deformation}$$

## Methods

Among the various computational methods available, computational fluid dynamics (CFD) and FSI are commonly employed for calculating fluid pressure, specifically ICP^22,25,27,30,31^. However, when dealing with deformable boundaries between the CSF and the brain, particularly within the inner brain layer, it becomes evident that CFD is an inadequate simulation method for ICP calculations, as noted by Gholampour et al^25^. Consequently, in the present study, we utilized a two-way FSI approach with strong coupling based on arbitrary Lagrangian–Eulerian formulations, employing ADINA software version 9.6, to calculate ICP and consequently ICC and brain stiffness values based on Eqs. 1 and 2.

### Patient population

**Figure 1:**
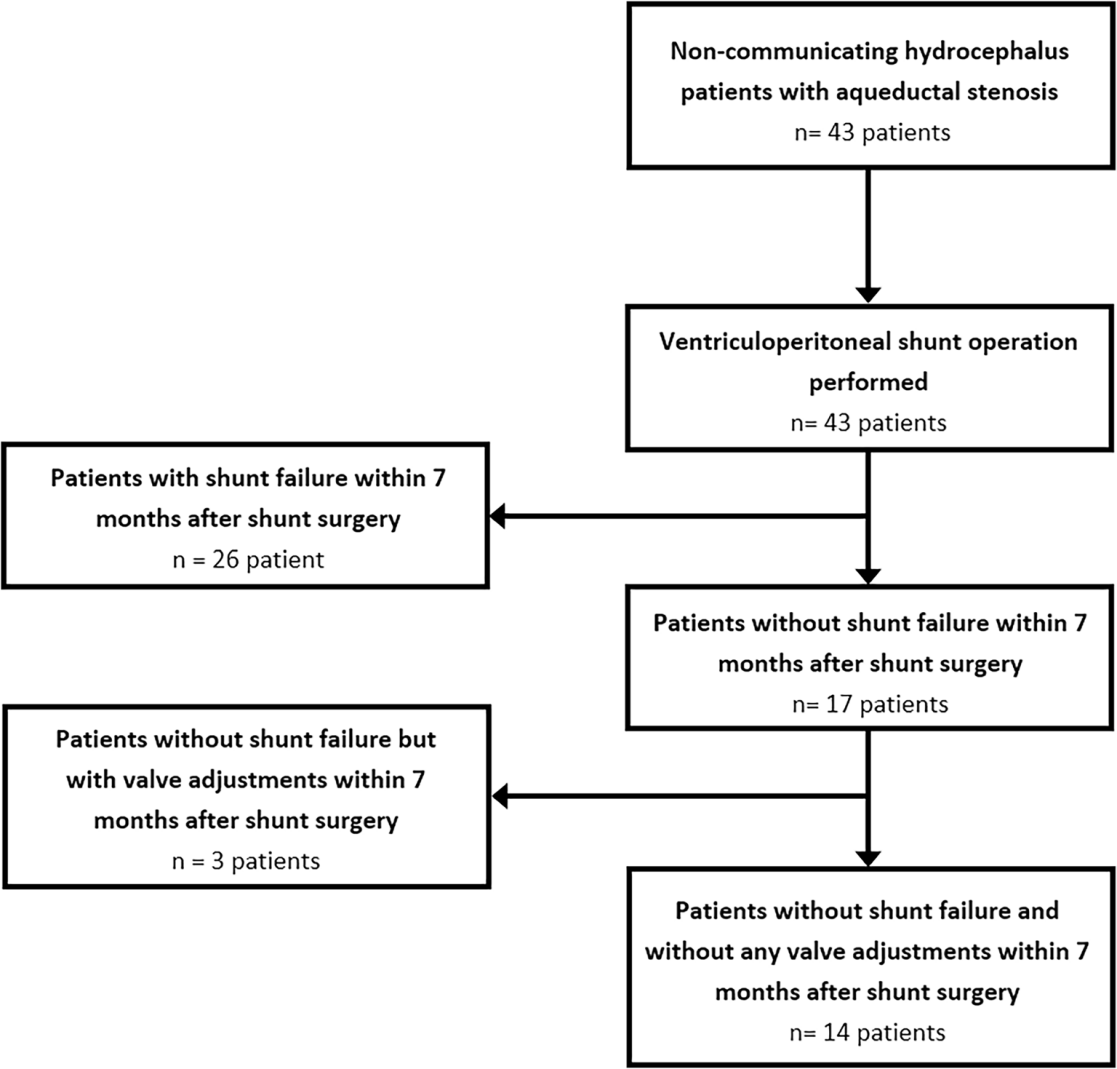
Study population selection. Flowchart illustrating the selection process of the study population.

Out of the initial 43 adult non-communicating hydrocephalus patients with aqueductal stenosis, we selected 14 patients who demonstrated improved outcomes after shunt surgery without requiring adjustments to the valve pressure setting over the 7-month post-surgery period (Fig. 1). Therefore, the calculated ICPs in this study are the result of natural interactions among blood, the brain, and CSF without manipulating and changing shunt valve pressure settings. All patients underwent treatment with a Medtronic ventriculoperitoneal shunt. Head cine phase-contrast (CINE PC) MRI was conducted at eight stages: one stage before the shunting procedure, and then at 1, 2, 3, 4, 5, 6, and 7 months following the shunting procedure. Because our previous studies indicated that CSF dynamic parameters generally stabilize 6–9 months after shunt surgery, further follow-up is unnecessary^18^. The body mass index of the patients was 25.2–28.6 kg/m^2^, and the age of the patients was 28–59 years (57.1% women and 42.9% men). The study design, protocols, and procedures were approved by the Human Institutional Review Board committee of the University of Chicago and the Tarish Hospital Research Ethics Board, adhering to the ethics guidelines of both institutions and following the 1964 Helsinki Declaration and its subsequent amendments. It should be noted that patient data was anonymized, and informed consent was obtained from all participants to ensure adherence to ethical research standards.

### CINE PC MRI setting

MRI encompassed cardiac-gated PC imaging to measure CSF velocity, along with axial T2 weighted imaging (T2WI). The parameters for axial T2WI consisted of echo and repetition times of 117 ms and 4,000 ms respectively, a field of view of 220 × 220 mm, slice intervals/slice thickness of 1.8 mm/6 mm, and a flip angle of 90°. For PC-MRI, the corresponding values were 7 ms and 21 ms for echo and repetition times, 160 × 160 mm for the field of view, 1.2 mm/6 mm for slice intervals/slice thickness, and 10° for the flip angle. The acquisition times for axial T2WI and PC-MRI were 150 s and 270 s, respectively. It should be noted that for the PC-MRI sequence, the imaging slices were carefully positioned to transect the anterior horn of the lateral ventricle, which is crucial for accurately assessing the CSF inlet flow dynamics. Velocity encoding in PC-MRI was set at 15 cm/s. The MRI data was generated using a 3 Tesla MRI system (Magnetom Trio, Siemens Erlangen, Germany).

### FSI simulation

We utilize FSI simulation to compute ICP, which serves as the denominator of the ICC equation (Eq. 1 ) and numerator of the brain stiffness equation (Eq. 2). In our two-way FSI computational model, CSF is treated as an incompressible Newtonian fluid, and brain tissue is defined as a solid domain. The study incorporates the law of continuity for CSF production in the ventricular system (Eq. 3) and subarachnoid space (SAS) (Eq. 4) coupled with the Navier–Stokes equation (Eq. 5) as the equations relevant to the fluid domain (CSF). In contrast to the ventricular system, it is important to emphasize that there is no CSF production in the SAS (Eq. 4)^22,25,31^. The behavior of the solid domain (brain) is governed by Eq. 6.

3 $$\nabla .{\varvec{u}}_{F} = S$$

4 $$\nabla .{\varvec{u}}_{F} = 0$$

5 $$\rho_{F} \frac{{\partial {\varvec{u}}_{{\varvec{F}}} }}{\partial t} + \rho_{F} (\left( {{\varvec{u}}_{F} - {\varvec{W}}).\nabla } \right){\varvec{u}}_{F} = - \nabla {\text{p}} + \mu \nabla^{2} {\varvec{u}}_{F} + {\varvec{f}}_{F}^{{\varvec{B}}}$$

6 $$\nabla .{\varvec{\sigma}}_{{\varvec{S}}} + {\varvec{f}}_{F}^{{\varvec{B}}} = \rho_{S} \mathop {{\varvec{u}}_{S} }\limits$$

The velocities of CSF and the moving mesh are represented by ${\varvec{u}}_{F}$ and ${\varvec{W}}$ individually. The densities of the brain and CSF are indicated by $\rho_{F}$ and $\rho_{S}$, respectively. CSF pressure and dynamic viscosity are denoted by ${\text{p}}$ and $\mu$. The variable $S$ represents CSF production in the ventricles, while ${{\varvec{f}}}_{C}^{{\varvec{B}}}$ signifies the body force per unit volume. In the governing equation for the brain, $\ddot{{{\varvec{u}}}_{S}}$ signifies local acceleration, and ${{\varvec{\sigma}}}_{{\varvec{S}}}$ refers to the stress within the brain tissue. The dynamic viscosity and density of CSF are assumed to be 0.001 kg.m^−1^.s^−1^ and 998.2 kg.m^−3^, respectively^18,22,24,25^.

Previous studies suggested the poro-viscoelastic constitutive model for human brain tissue^32^. This model is shown to be highly accurate for hydrocephalic brains and has been validated against experimental data^22,33,34^. Elkin et al. demonstrated that the most accurate alignment with experimental results occurs when the brain's viscoelastic component in the poro-viscoelastic model is represented through the Prony series for shear modulus ($G_{r} \left( t \right)$)^35^. Consequently, we incorporated this approach to incorporate the time parameter (*t*) in our poro-viscoelastic brain model, as specified in Eq. 7.

7 $$G_{r} \left( t \right) = {\text{G}}_{0} \left( {1 - \mathop \sum \limits_{k = 1}^{N} g_{k}^{p} \left( {1 - e^{{ - \frac{t}{{\tau_{k} }}}} } \right)} \right)$$

where $\tau$_k_, ${\text{G}}_{0}$, and $g_{k}^{p}$, are relaxation time, input shear modulus, and relaxation modulus, respectively. In our study, we utilize specific parameters for the Prony series, which include $\tau_{1}$, $\tau_{2}$, $\tau_{3}$, and $g_{k}^{p}$, set at 3.1 s, 27 s, 410 s, and 0.285, respectively^18,22,24,25,33,36^. Additionally, the study utilizes constant values for elasticity, permeability, Poisson's ratio, and void ratio, which have been validated through previous research^21,22,33,36^. These validated values that we have employed are 584.4 Pa, 4.08 × 10–12 M^4^/N.s, 0.35, and 0.2, respectively.

### Boundary conditions

**Figure 2:**
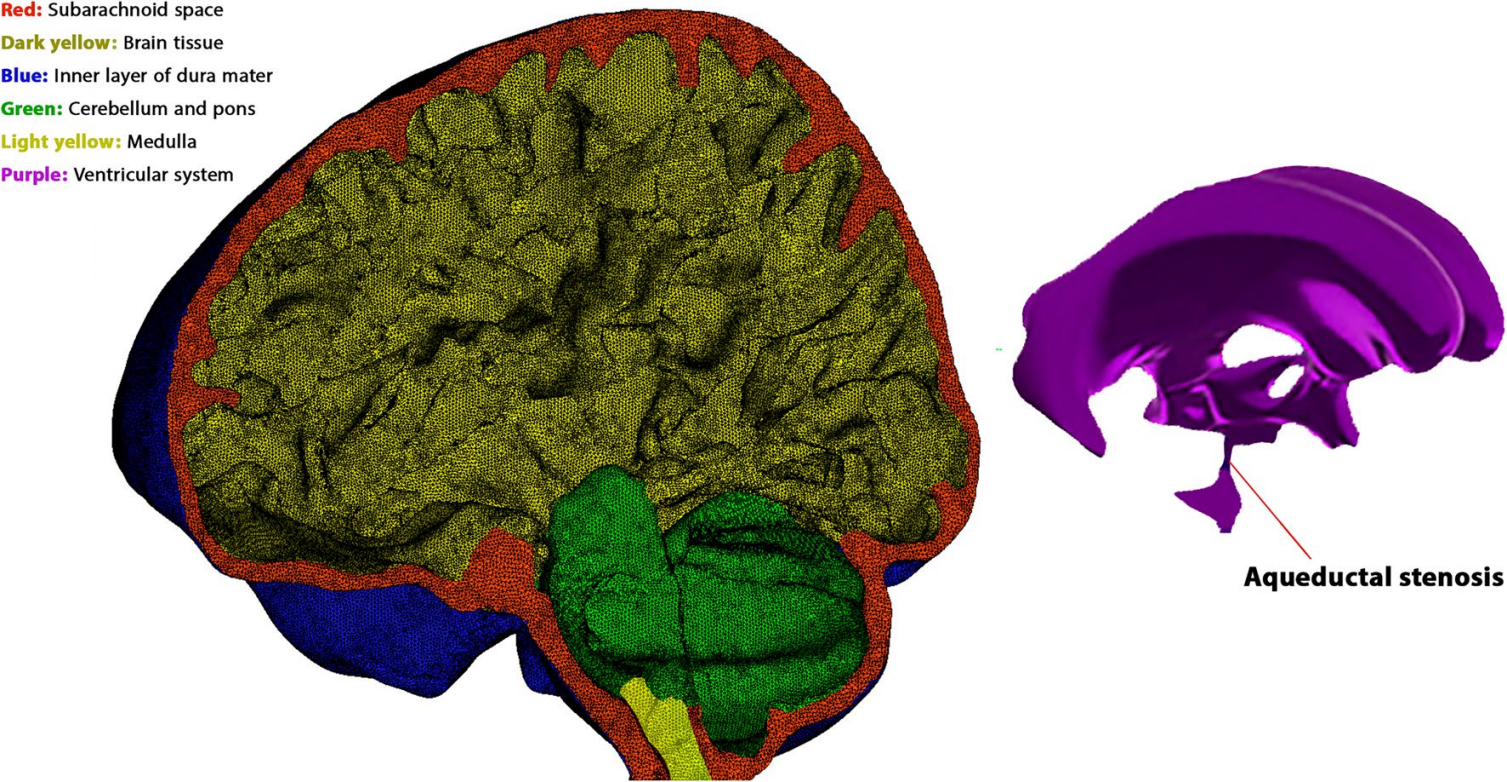
Geometrical model and mesh generation. 3-D geometrical meshed model of head substructures (except for ventricles) and 3-D geometrical model of the ventricular system for a non-communicating hydrocephalus patient before shunt surgery.

**Figure 3:**
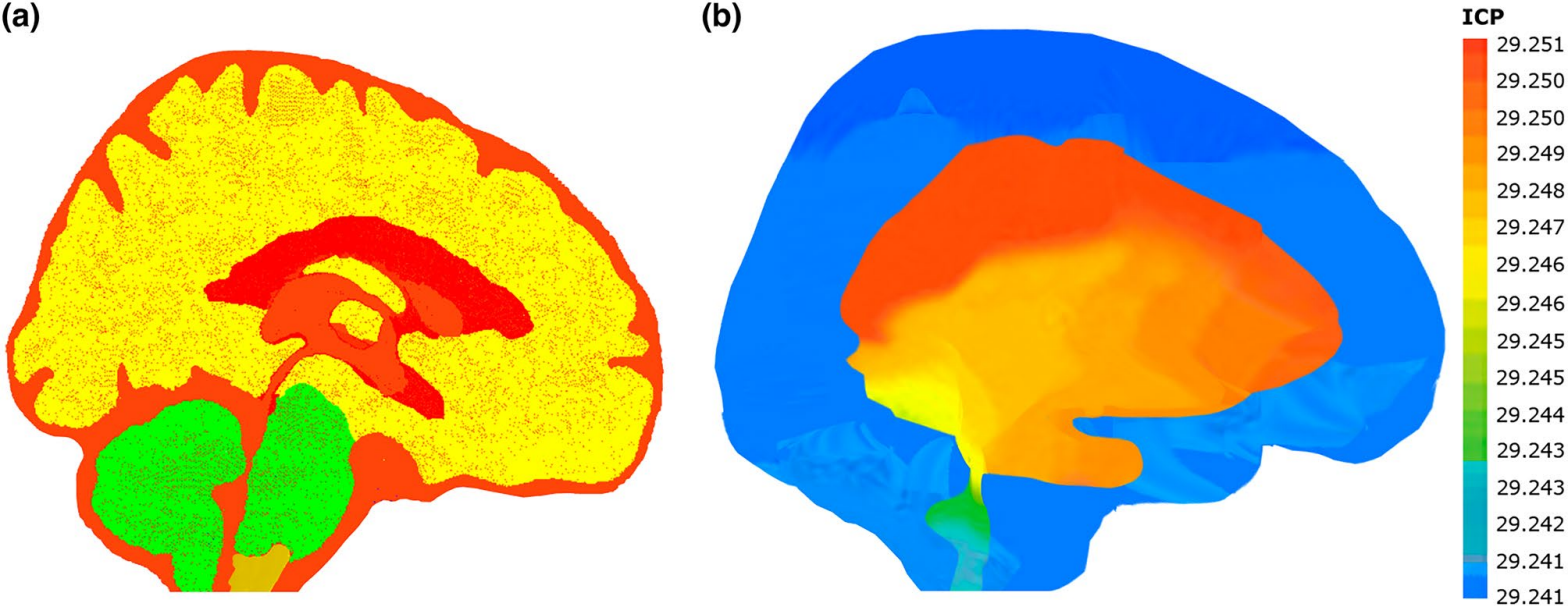
Geometrical boundary conditions and FSI simulation result. **(a)** Geometrical boundary conditions for a treated non-communicating hydrocephalus patient 7 months after shunt surgery. The red color represents the fluid domain, while other colors represent the solid domain. The interfaces between the red and yellow colors are defined as FSI boundaries. **(b)** Calculated ICP using FSI simulation for a patient before shunt surgery. The unit of ICP is cmH_2_O. Fluid-structure interaction (FSI); Intracranial compliance (ICP).

The FSI simulation's accuracy is highly dependent on the choice of boundary conditions^25^. Our previous study demonstrates that for hydrocephalus patients, the most accurate inlet and outlet boundary conditions are achieved using pulsatile CSF flow rate^22,25^. We created 3D geometrical models of the head substructures by employing image reconstruction techniques to be used as geometric inputs for the FSI simulation (Fig. 2). Then we measured the intracranial volume and brain surface area from these 3D geometrical models, representing the numerator of Eqs. 1 and 2 for the calculation of ICC and FSI-based brain stiffen. It's worth noting that the 3-D geometrical model used in this study includes the CSF model (ventricular system and SAS) and solid model (Figs. 2 and 3a). Besides the geometric input, we incorporated CSF dynamic inputs for the FSI simulation. The largest production of CSF happens in the lateral ventricles^37^. Hence, for FSI simulation purposes, the frontal horns of the lateral ventricles are considered the dynamic inlet flow location^22,25,31^. On the other hand, the spinal cord and sagittal sinus are designated as the outlets^18,22,24,25^. The study utilizes inlet/outlet boundary conditions derived from CSF flow rate graphs that combine a constant value graph and a pulsatile graph, based on our previous finding^25^. The constant graph for the CSF inflow at the inlet, CSF outflow in the spinal cord, and CSF outflow in the sagittal sinus were 0.35, 0.17, and 0.18 ml/min, respectively^25,37–39^. The pulsatile graphs are obtained from in vivo measurements of CSF flow rates in anterior horn of lateral ventricle using CINE PC MRI. These inlet and outlets boundary graphs are calculated separately for each patient at each stage using MATLAB software (version R2018; Mathworks, Natick, MA, USA) and applied in ADINA software during the FSI simulation process.

Previous studies have determined that the skull and dura mater do not significantly affect ICP calculations for hydrocephalus patients^25^. Consequently, the inner layer of the dura mater is constrained by a no-slip boundary condition (Figs. 2 and 3a). Interfaces between the inner and outer layers of the brain with CSF are defined as FSI boundaries (Fig. 3a). These boundaries are governed by displacement compatibility, traction equilibrium, and velocity matching equations, as described in Eqs. 8, 9, and 10, respectively. These equations apply to the FSI interfaces and ensure proper interactions between CSF and brain tissue.

8 $${\varvec{d}}_{S} = {\varvec{d}}_{F} \user2{ }\left( {x, y, z} \right)\varepsilon \;\Gamma_{wall}^{F} \cap \Gamma_{wall}^{S}$$

9 $${\varvec{\sigma}}_{S} .{\varvec{n}} = {\varvec{\sigma}}_{F} .{\varvec{n}} \left( {x, y, z} \right)\varepsilon \;\Gamma_{wall}^{F} \cap \Gamma_{wall}^{S}$$

10 $${\varvec{u}}_{S} = {\varvec{u}}_{F} \left( {x, y, z} \right)\varepsilon \;\Gamma_{wall}^{F} \cap \Gamma_{wall}^{S}$$

The displacements of the brain and CSF, introduced along the FSI boundaries, are represented by ${\varvec{d}}_{S}$ and ${\varvec{d}}_{F}$. In this context, ${\varvec{\sigma}}_{S} .{\varvec{n}}$ and ${\varvec{\sigma}}_{F} .{\varvec{n}}$ represent the stress tensors of the brain and CSF, respectively, oriented in the normal direction of the FSI interfaces. The local acceleration of the brain is denoted as ${{\varvec{u}}}_{S}$. It is also important to emphasize that the meshing of the models, prior to the application of boundary conditions, employed tetrahedral (four-node) elements (Fig. 2).

As we said, we only recruited patients with improved outcomes and without valve adjustments over 7 months after shunt surgery. Hence, we defined an outlet pressure instead of the shunt, as reported in the Medtronic shunt catalog^40^. After applying the abovementioned processes, we calculated the ICP using FSI simulation for all 14 patients before shunt surgery and 7 stages after shunt surgery (Fig. 3b) to be used for ICC and FSI-based brain stiffness calculation based on Eqs. 1 and 2.

### Experimental ICP measurement setting

To validate the simulated ICP calculated through the FSI method, we compared them with experimental ICP measurements. To enable accurate monitoring of ICP, cranial perforations with a diameter of 2.2 mm were carefully created in the skulls of ten patients scheduled for shunt surgery. Subsequently, we inserted an ICP micro-sensor (Codman MicroSensor, Johnson and Johnson) to a depth of 1.5–2 cm. It is noteworthy that before introducing the needle, the sensor was calibrated to atmospheric pressure.

### Statistical analysis

Statistical analysis was also conducted using IBM SPSS software, version 20.0, IBM Corp, Armonk, NY, USA. It should be noted that both the ICC and brain stiffness datasets exhibited a normal distribution according to the Shapiro–Wilk test. Hence, both the Pearson and Spearman correlation coefficients were employed to analyze the relationship between ICC and FSI-based brain stiffness. Statistical significance was considered at a threshold of *P*-value < 0.05.

## Results and discussion

### Data validation

**Figure 4:**
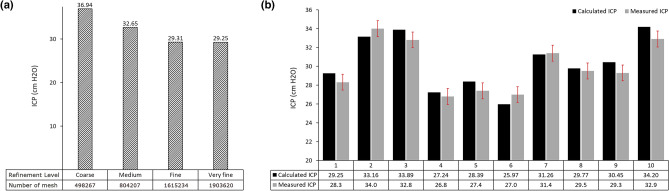
Grid independence study and data validation. **(a)** A comparative analysis of ICP utilizing various mesh densities—coarse, medium, fine, and very fine. **(b)** Comparison of measured ICP using the experimental ICP monitoring method and calculated ICP using the FSI method for hydrocephalus patients before shunt surgery. Intracranial pressure (ICP).

We explored the impact of grid refinement on ICP calculations, providing valuable insights into numerical accuracy and simulation efficiency. A grid independence study confirmed the convergence of the mesh, demonstrating negligible differences between fine and very fine meshes (less than 0.24%) (Fig. 4a). Regarding the calculation of ICC and FSI-based brain stiffness through FSI simulation, it is imperative to ensure the accuracy of our simulated results using data validation. This is because data validation stands as a paramount concern within computer simulation projects. It should be noted that other terms of Eqs. 1 and 2, except for ICP, were directly measured from the 3-D models. Consequently, the only source of potential error in the calculation of ICC and FSI-based brain stiffness could be ICP. Therefore, we experimentally measured ICP using the ICP monitoring method for only 10 patients, specifically before the shunt surgery stage, due to medical limitations and the recommendation of neurosurgeons. Then we compared these measured ICP values with the corresponding ICP calculated using FSI simulation. The data validation results showed that the differences between calculated and measured ICP were less than 3.9% (Fig. 4b). The results from Supplementary Fig. 1 also demonstrated a robust relationship between measured and calculated ICP, with *p*-values of 0.000 for both Pearson and Spearman correlation analyses, and corresponding coefficients of 0.95 and 0.94, respectively.

### Correlation between FSI-based brain stiffness and ICC

**Figure 5:**
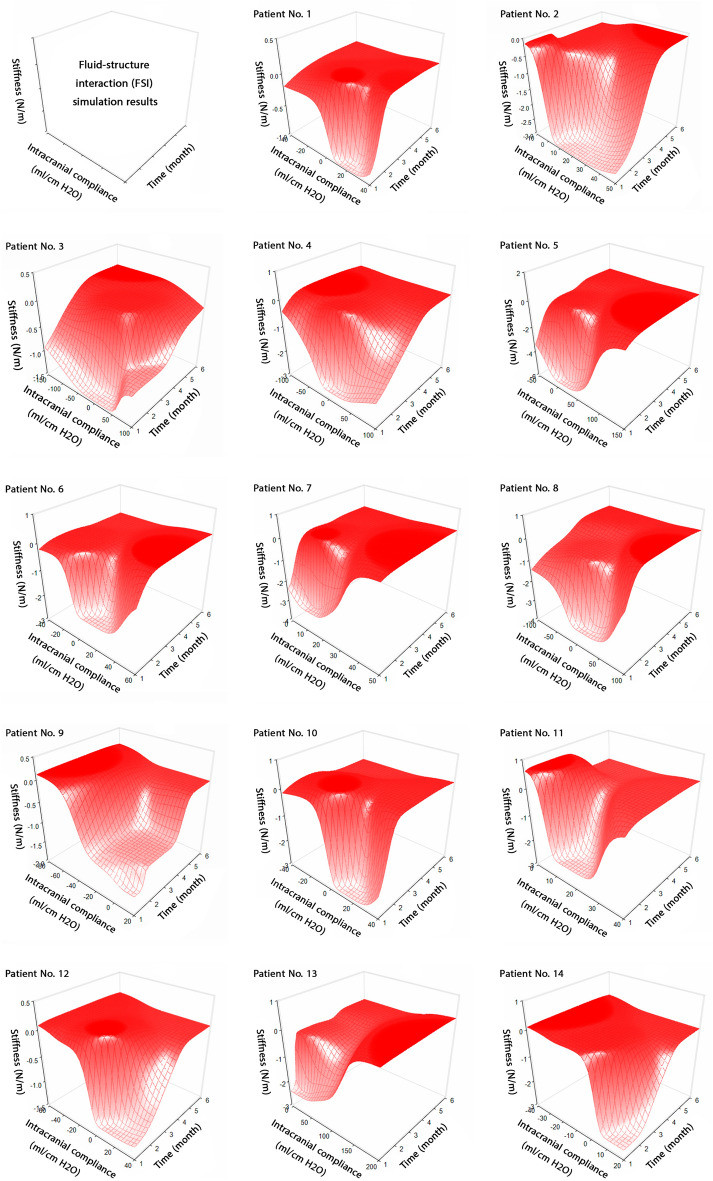
Changes in ICC and FSI-based brain stiffness. Concurrent changes in ICC and FSI-based brain stiffness over 6 months after shunt surgery. The surfaces of graphs in the 14 patients were nonuniform, reflecting variations and changes related to the oscillatory behavior of ICC and FSI-based brain stiffness over an extended duration. The negative values of ICC indicate variations in ICC changes over time, as observed in the study by Okon et al^72^. Positive and negative stiffness values reflect increases and decreases in brain size due to these variations. The Raw data for Fig. 5 is included in Supplementary Table 1. Fluid–structure interaction (FSI).

**Figure 6:**
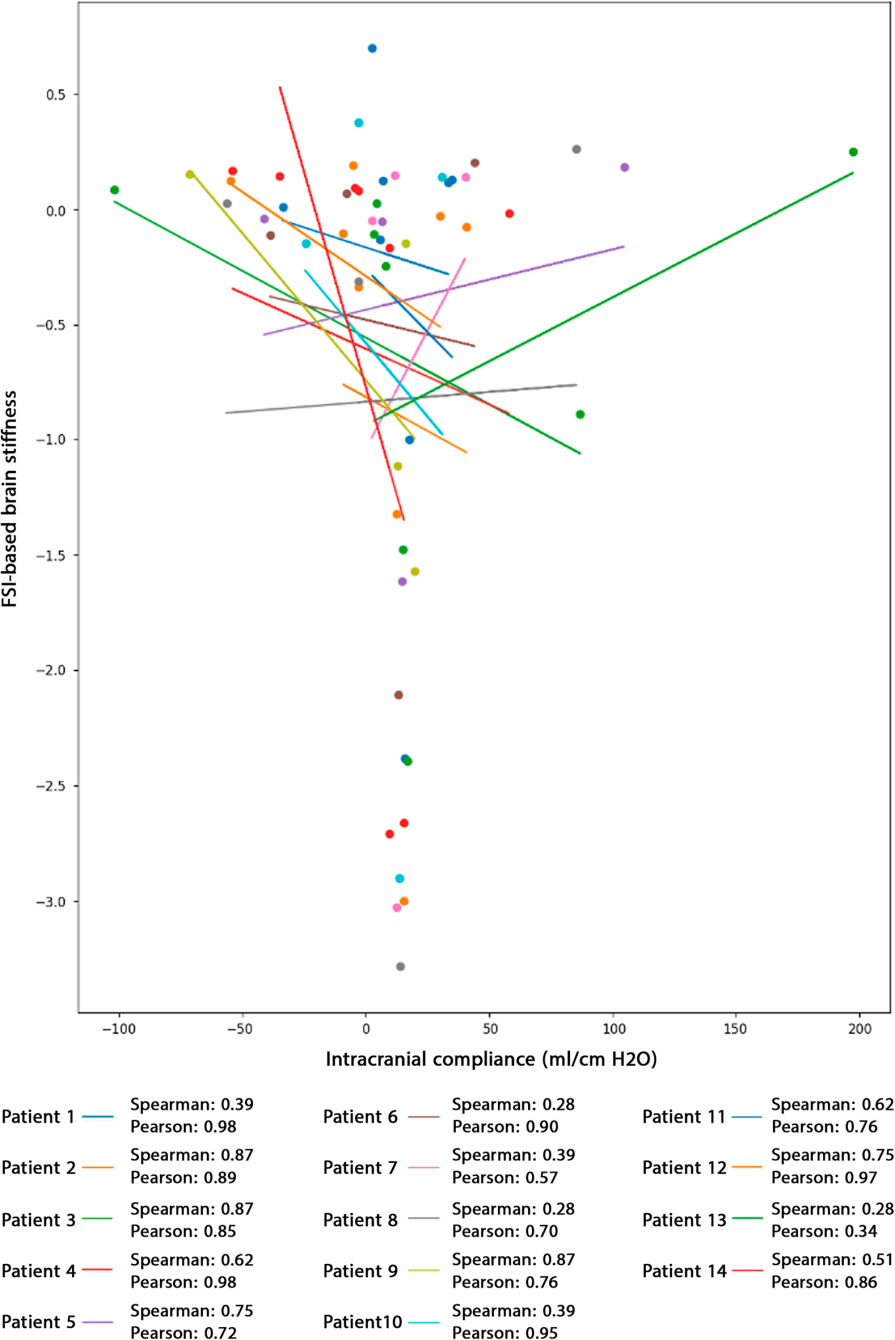
Correlation between ICC and FSI-based brain stiffness. The Pearson correlation coefficient between ICC and FSI-based brain stiffness was computed for all 14 patients. The correlation analysis revealed no significant relationship between FSI-based brain stiffness and ICC. It should be noted that the figure displays Pearson correlation results; however, the *p*-values for both Pearson and Spearman correlations are listed at the bottom of the figure. The negative values of ICC indicate variations in ICC changes over time, as observed in the study by Okon et al^72^. Positive and negative stiffness values reflect increases and decreases in brain size due to these variations. Raw data for Fig. 6 is included in Supplementary Table. Fluid–structure interaction (FSI); Intracranial compliance (ICC).

Figure 5 illustrates changes in FSI-based brain stiffness and ICC in hydrocephalus patients over six months after shunt surgery. Notably, the differences between the parameters at the 6 and 7-month intervals were not substantial; therefore, the values of ICC and FSI-based brain stiffness at the 7-month interval have not been reported. Although this project focused solely on patients with improved outcomes and no changes in valve performance levels after shunt surgery, there were observed oscillatory variations in the parameters following the shunt surgery (Fig. 5). The correlation analysis presented in Fig. 6 reveals no relationship between FSI-based brain stiffness and ICC across our cohort of 14 patients. Despite our initial hypothesis, the Pearson and Spearman correlation analysis revealed no statistically significant correlation between these two parameters. This may underscore the multifaceted nature of intracranial dynamics that are influenced by factors beyond the mechanical properties of brain tissue alone. ICC is intricately linked with CSF dynamics, cerebral perfusion pressure (CPP), and the mechanics of cerebral blood vessels. These factors collectively influence the brain's biomechanical environment, which may not be directly reflected by measurements of brain stiffness alone. For instance, variations in CSF dynamics and CPP can alter ICP and, thereby, ICC, without a direct correlation to the stiffness of brain tissue. Similarly, changes in the mechanics of cerebral blood vessels, influenced by systemic factors or localized vascular pathology, might impact ICC independently of brain tissue stiffness.

### Feasibility of ICC assessment using MRE-based brain stiffness

**Figure 7:**
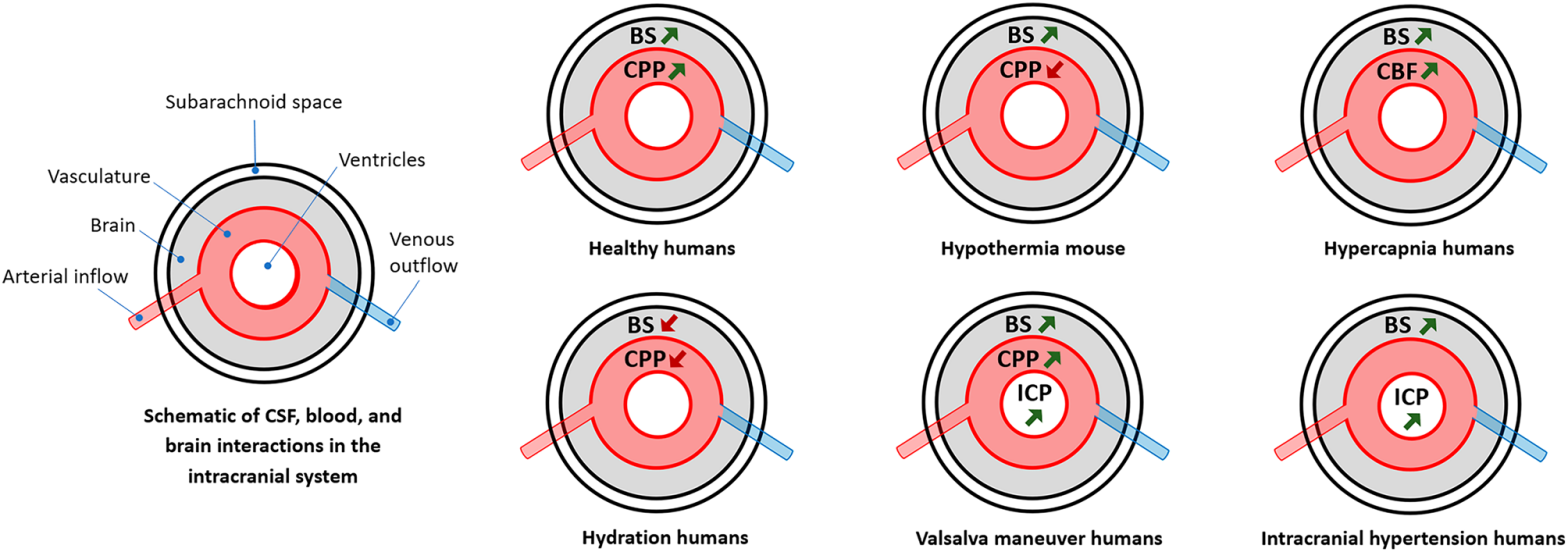
Variations in MRE-based brain stiffness with cerebral perfusion pressure (CPP), cerebral blood flow (CBF), and intracranial pressure (ICP). Changes in MRE-based brain stiffness (BS) lead to alterations in cerebral perfusion pressure (CPP), cerebral blood flow (CBF), and intracranial pressure (ICP) in patients with hypothermia, hypercapnia, hydration, and intracranial hypertension, as well as in healthy subjects and healthy subjects undergoing the Valsalva maneuver. The arrows in the figure illustrate how these alterations occur, whether through direct or indirect relationships.

Despite the aforementioned findings indicating an absence of correlation between ICC and FSI-based brain stiffness, it is essential to distinguish between the methodologies used to assess brain stiffness. Previously, we differentiated between FSI-based calculation and MRE-based measurement of brain stiffness. This distinction is critical, as each method offers unique insights into the mechanical properties of brain tissue, potentially influencing the observed relationship (or lack thereof) with ICC. Therefore, exploring the potential relationship between ICC and brain stiffness as measured by MRE is of significant importance. There is a substantial body of literature in the fields of non-linear viscoelastic dynamics and poroelasticity indicating that MRE-based brain stiffness is sensitive to changes in ICP^32,41–45^. Figure 7 demonstrates the variation and sensitivity of brain stiffness, as measured by MRE and ultrasound elastography, to blood and CSF dynamic parameters such as CPP, CBF, and ICP in subjects with various conditions, including hypothermia (as observed in a mouse study)^46^, hypercapnia^47^, hydration^48^, and intracranial hypertension^49^, as well as in healthy subjects^50^ and healthy subjects undergoing the Valsalva maneuver^51^. It is essential to acknowledge that variations in brain stiffness in response to changes in CPP, CBF, and ICP do not consistently exhibit a direct relationship. To clarify the nature of these variations—be they direct or inverse—arrows have been employed in Fig. 7. These findings may address the concerns regarding the lack of a relationship between brain stiffness and CSF and blood dynamics, which significantly influence ICC alterations. However, some studies raise another concern related to the insufficient ability of MRE to account for factors such as mechano-vascular effects that are important and effective in ICC assessment^52^. Forouhandehpour et al. attempted to address this concern^53^. They used functional intrinsic MRE to investigate intrinsic vascular mechanical reactions during visual stimulation and found a relationship between vasodilation and MRE-based stiffness changes. Following vasodilation, they also observed a significant reduction in stiffness around the primary visual cortex region, particularly in areas adjacent to the posterior cerebral artery.

There are also other concerns that are necessary to be considered for the assessment of ICC through MRE-based brain stiffness. Our recent study underscored the significance of the behavior and response of the brain to loading due to brain disorders, specifically hydrocephalus^24^. Therefore, understanding the behavior and response of the brain is crucial for comprehending the relationship between MRE-based brain stiffness and ICC. When the loading condition on the brain changes due to various brain disorders, an initial zone of ICC change occurs, where the brain strives to maintain stable ICP despite volume changes^54,55^. This behavior and response are connected to compensatory mechanisms, where veins assist in draining deoxygenated blood to regulate volume and maintain pressure. Subsequently, a late zone of ICC change occurs when the brain's compensatory mechanisms become depleted, resulting in an elevation of ICP as volume continues to increase. Another concern is the complex pore structures of the brain. The brain consolidation theory underscores the significance of the brain's pore structure. This theory explains that when the brain experiences load changes due to brain disorders, the pore structure undergoes deformation^56^. This deformation triggers fluid displacement within the pores, leading to changes in the brain's viscous component, resulting in changes in CSF dynamics and ICC^56,57^. Brain MRE can also quantify changes in the brain's viscous component in addition to the elastic component by measuring the attenuation of externally induced shear waves^58,59^. This may highlight MRE's discriminative potential as a diagnostic tool, as evidenced by Table 1, which demonstrates meaningful differences in brain stiffness—encompassing both viscous and storage moduli—between healthy subjects and patients with brain disorders such as multiple sclerosis, Alzheimer's disease, brain tumors, and Parkinson's disease.

**Table 1:** MRE-based brain stiffness values based on different brain regions and frequencies for adult patients with brain disorders and healthy subjects.

Health Status	Author, year	Age	Brain region	Frequency (Hz)	Stiffness (kPa)	Source
Parkinson's disease	Lipp et al. ^60^	18 Adult patients	Whole brain	25–62.5	1.88 ± 0.26	^60^
			Lentiform nucleus	25–62.5	1.96 ± 0.21	
Normal pressure hydrocephalus	Perry et al. ^61^	10 Adult patients	Cerebrum	60	2.64 ± 0.11	^61^
			Frontal lobes	60	2.65 ± 0.05	
			Occipital lobes	60	2.97 ± 0.15	
			Parietal lobes	60	2.63 ± 0.18	
			Temporal lobes	60	2.79 ± 0.48	
			Deep gray	60	2.91 ± 0.09	
			Cerebellum	60	2.20 ± 0.04	
			Periventricular	60	1.74 ± 0.24	
	ElSheikh et al. ^7^	20 Adult patients	Cerebrum	60	2.46 ± 0.08	^7^
			Frontal lobes	60	2.47 ± 0.10	
			Occipital lobes	60	2.67 ± 0.13	
			Parietal lobes	60	2.46 ± 0.17	
			Temporal lobes	60	2.63 ± 0.12	
			Deep gray/white matter	60	2.58 ± 0.23	
			Sensorimotor cortex	60	3.12 ± 0.41	
			Cerebellum	60	2.08 ± 0.10	
Alzheimer’s disease	ElSheikh et al. ^7^	8 Adult patients	Cerebrum	60	2.32 ± 0.09	^7^
			Frontal lobes	60	2.36 ± 0.10	
			Occipital lobes	60	2.54 ± 0.20	
			Parietal lobes	60	2.26 ± 0.09	
			Temporal lobes	60	2.52 ± 0.09	
			Deep gray/white matter	60	2.48 ± 0.24	
			Sensorimotor cortex	60	2.46 ± 0.10	
			Cerebellum	60	2.05 ± 0.13	
	Murphy et al. ^62^	7 Adult patients	Whole brain	50 and 90	2.20	^62^
Brain tumor	Streitberger ^63^	18 Adult patients	Tumor (region of interest)	30–60	1.3	^63^
			White matter (reference)	30–60	1.80	
	Simon et al. ^64^	16 Adult patients	Tumor (region of interest)	45	1.40	^64^
			White matter (reference)	45	1.83	
Healthy controls	Lipp et al. ^65^	12 Adult subjects	Whole brain	30–60	1.04 ± 0.08	^65^
			Frontal lobes	30–60	1.15 ± 0.21	
			Striatum	30–60	1.24 ± 0.33	
			Caudate	30–60	0.79 ± 0.17	
			Thalamus	30–60	0.91 ± 0.15	
			Mesencephalic	30–60	0.96 ± 0.08	
	Gerischer et al. ^66^	21 Adult subjects	Hippocampus	–	1.08 ± 0.19	^66^
			Thalamus	–	1.28 ± 0.20	
			White matter	–	1.54 ± 0.13	

Despite optimism regarding the capabilities of MRE-based measurements of brain stiffness, concerns persist regarding their applicability across all types of brain disorders. Our recent study has differentiated brain disorders into two categories: gradual-onset and acute-onset^20^. Gradual-onset disorders, such as primary hydrocephalus or Alzheimer’s disease, exhibit symptoms that develop gradually, reflecting long-term load on the brain. In contrast, acute-onset disorders, such as those resulting from traumatic brain injury due to concussion, present symptoms rapidly following an immediate load. A critical distinction between these types of disorders relates to the brain tissue's response to the removal of a load associated with the disorder. We have raised significant doubts regarding the efficacy of MRE-based brain stiffness measurements as a diagnostic tool for gradual-onset disorders^67^. Our recent study emphasizes the profound long-term effects of the viscous component of brain tissue on material property changes in disorders like hydrocephalus, observed over two months^18,24^. Furthermore, our findings indicated that both the duration of load application and the strain rate—parameters that are notably significant in gradual-onset disorders—markedly affect brain stiffness, recovery trajectory, and treatment efficacy. However, there are challenges in adapting MRE protocols to accommodate these factors^67^. Extending the acquisition time to capture prolonged effects could inadvertently result in an overestimation of brain stiffness due to potential overshooting stiffening responses^67^. Therefore, it is more logical to propose the use of MRE-based brain stiffness measurements for non-invasive evaluation of ICC in patients with acute-onset brain disorders.

### Limitations and future directions

Our findings offer an avenue for the non-invasive assessment of ICC using MRE-based brain stiffness, particularly in patients with acute-onset brain disorders. Nonetheless, future studies should involve measuring ICC and MRE-based brain stiffness in human subjects and patients with different brain disorders to definitively confirm the hypothesis that brain MRE can effectively assess ICC. This is crucial because aside from CSF and blood dynamics, other factors such as interstitial fluid dynamics are also associated with ICC changes. Several studies have shown a decline in glymphatic drainage and ICC values in patients with NPH, Alzheimer's disease, and traumatic brain injury^68–70^. Therefore, future investigations are necessary to determine whether MRE-based stiffness can consider the effects of all related parameters with ICC, such as interstitial fluid dynamics and glymphatic drainage in all brain disorders. One of the challenges in accurately visualizing and comparing differences in brain stiffness between healthy individuals and patients with brain disorders stems from the use of varying vibration frequencies across studies. Stiffness is known to be significantly influenced by the frequency of vibration, making direct comparisons complex^71^. It is recommended that future research efforts report stiffness values as a function of excitation frequency to facilitate a more nuanced analysis of brain stiffness disparities between healthy and diseased states^71^. Consequently, this approach could enhance our comprehension of the variations in MRE-based stiffness and ICC relationships between healthy individuals and patients with brain disorders.

## Conclusion

Gaining insight into the relationship between ICC and brain stiffness, as well as understanding the contribution and potential of brain MRE in assessing ICC based on brain stiffness, can be valuable due to the clinical importance of ICC and the non-invasive nature of the MRE technique. This study used a validated computational method to calculate ICP, forming the foundation for ICC and FSI-based brain stiffness in hydrocephalus patients with aqueductal stenosis. The findings revealed no correlation between ICC and FSI-based brain stiffness. However, the results demonstrated optimism regarding the assessment of ICC based on MRE-based brain stiffness in patients with acute-onset brain disorders. Future studies will require additional investigation to better understand the direct relationship between them, enabling the practical use of MRE-based brain stiffness, and enhancing our diagnostic capabilities in the realm of acute-onset brain disorders.

### Supplementary Information

Supplementary Information.

## Data Availability

All data used in this manuscript are publicly available and can be found in original publications or repositories. The MRI files of subjects, however, contain some identifying information about patients and normal subjects, and cannot be made publicly available. The data are available from the corresponding author.

## Abbreviations

ICC: Intracranial compliance
CSF: Cerebrospinal fluid
ICP: Intracranial pressure
MRE: Magnetic resonance elastography
FSI: Fluid–structure interaction
MRI: Magnetic resonance imaging
CPP: Cerebral perfusion pressure
CBF: Cerebral blood flow
PC: Phase-contrast
T2WI: T2 weighted imaging

## Code availability

The codes and simulation process setting are available upon request to corresponding author.
