# Supplementary material for: Feasibility of assessing non-invasive intracranial compliance using FSI simulation-based and MR elastography-based brain stiffness
Source: Sci Rep. 2024 Mar 18;14:6493. doi: 10.1038/s41598-024-57250-4 (PMC10948846; doi:10.1038/s41598-024-57250-4)
Supplement: Supplementary file 1 — Supplementary Information. [file 41598_2024_57250_MOESM1_ESM.docx]

**Feasibility of Assessing Non-Invasive Intracranial Compliance Using FSI Simulation-Based and MR Elastography-Based Brain Stiffness**

Seifollah Gholampour^1^

^1^ Department of Neurological Surgery, University of Chicago, Chicago, Illinois, USA.

***Correspondence:** Seifollah Gholampour, Ph.D.,

Department of Neurological Surgery

The University of Chicago

5841 S. Maryland Ave, 60637, Chicago, IL, USA.

**Tel:** (+1) 773-702-2123

**Fax**: (+1) 773-702-3518

**E-mail**: [seifgholampour@bsd.uchicago.edu](mailto:seifgholampour@bsd.uchicago.edu)

**ORCID:** 0000-0002-4924-4239

**Short running Title:** Brain Elastography and Intracranial Compliance

**Number of Figures:** 7

**Number of Table:** 1

**Number of supplementary Figure:** 1

**Number of supplementary Table:** 1

**Supplementary Figure 1.** The correlation analysis between measured and calculated ICP based on Pearson correlation analysis is depicted. The p-value for both Pearson and Spearman correlations was 0.000. The Pearson and Spearman coefficients were 0.95 and 0.94, respectively.


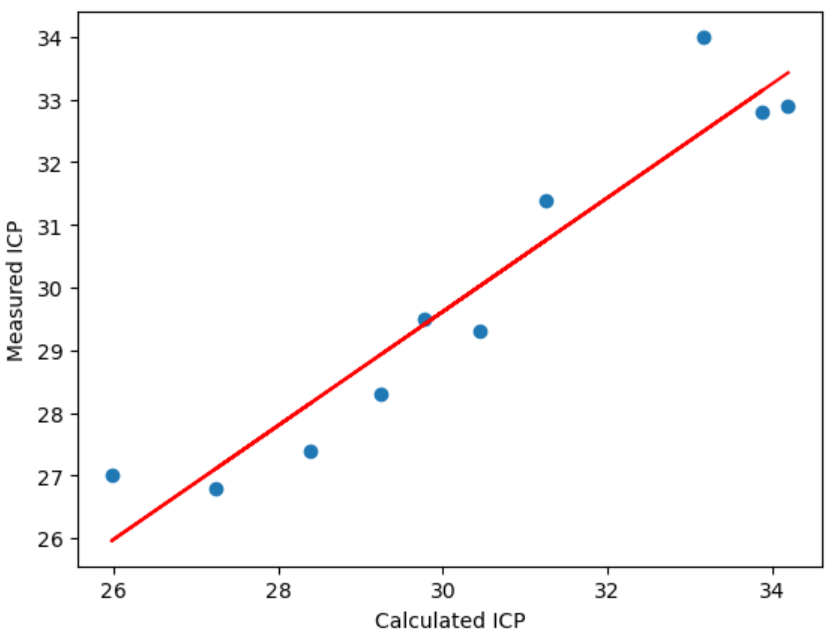


**Supplementary Table 1**. The calculated values for ICC and brain stiffness obtained through FSI simulation for 14 patients. Notably, the differences between the parameters at the 6 and 7-month intervals were not substantial and, therefore, have not been reported.

CSF: Cerebrospinal fluid; FSI: Fluid-structure interaction.

| Time | Patient number 1 | | Patient number 2 | | Patient number 3 | | Patient number 4 | | Patient number 5 | | Patient number 6 | | Patient number 7 | | Patient number 8 | | Patient number 9 | | Patient number 10 | | Patient number 11 | | Patient number 12 | | Patient number 13 | | Patient number 14 | |
| --- | --- | --- | --- | --- | --- | --- | --- | --- | --- | --- | --- | --- | --- | --- | --- | --- | --- | --- | --- | --- | --- | --- | --- | --- | --- | --- | --- | --- |
| Parameters  Elapsed time | ICC | Stiffness | ICC | Stiffness | ICC | Stiffness | ICC | Stiffness | ICC | Stiffness | ICC | Stiffness | ICC | Stiffness | ICC | Stiffness | ICC | Stiffness | ICC | Stiffness | ICC | Stiffness | ICC | Stiffness | ICC | Stiffness | ICC | Stiffness |
| 1-2 | 17.475 | -1.000 | 15.503 | -3.000 | 14.875 | -1.476 | 9.556 | -2.707 | 14.833 | -5.611 | 13.052 | -2.106 | 12.386 | -3.028 | 14.061 | -3.285 | 19.942 | -1.571 | 13.390 | -2.903 | 15.740 | -2.380 | 12.311 | -1.320 | 16.925 | -2.395 | 15.479 | -2.662 |
| 2-3 | 6.900 | 0.128 | -9.133 | -0.104 | 86.842 | -0.886 | -4.504 | 0.093 | 6.528 | -0.053 | -7.686 | 0.069 | 11.870 | 0.150 | -2.917 | -0.313 | 12.661 | -1.111 | -3.082 | 0.379 | 2.674 | 0.703 | -5.155 | 0.194 | 8.212 | -0.243 | -2.922 | 0.081 |
| 3-4 | 33.571 | 0.117 | -3.004 | -0.335 | 4.386 | 0.028 | -53.871 | 0.169 | 104.583 | 0.186 | 44.138 | 0.205 | 40.270 | 0.142 | 85.385 | 0.264 | -71.250 | 0.154 | 30.952 | 0.141 | 35.000 | 0.129 | -54.762 | 0.125 | 197.273 | 0.251 | -34.750 | 0.146 |
| 4-5 | -33.600 | 0.012 | 40.700 | -0.075 | -101.667 | 0.086 | 57.895 | -0.015 | -41.220 | -0.042 | -38.644 | -0.110 | 2.439 | -0.048 | -56.400 | 0.028 | 16.290 | -0.147 | -24.444 | -0.147 | 5.862 | -0.132 | 30.238 | -0.027 | 3.393 | -0.105 | 9.589 | -0.168 |
| 5-6 | 34.235 | 0.146 | 43.850 | -0.134 | 116.584 | 0.974 | 62.849 | 0.099 | 108.209 | -0.058 | 47.906 | 0.072 | 46.780 | 0.193 | 94.701 | 0.390 | 74.023 | 1.168 | 35.870 | 0.432 | 38.427 | 0.782 | 59.804 | 0.264 | 267.89 | 0.297 | 59.034 | 0.105 |
